# Immune repertoire sequencing for precision diagnosis in kidney transplantation

**DOI:** 10.1186/s12967-024-05229-0

**Published:** 2024-05-17

**Authors:** Lifei Liang, TingTing Chen, Tongyu Zhu, Cheng Yang

**Affiliations:** 1 Department of Urology, Zhongshan Hospital, Fudan University, No. 180 Fenglin Road, Shanghai, P. R. China; 2 Department of Pharmacy, Zhongshan Hospital, Fudan University, Shanghai, China; 3 Shanghai Key Laboratory of Organ Transplantation, Shanghai, China; 4 Zhangjiang Institue of Fudan University, Shanghai, China; 5 Shanghai Medical Collage, Fudan University, Shanghai, China

Rejection is a major challenge after kidney transplantation. Precision diagnosis is crucial for preserving the allograft function and longevity. While kidney allograft biopsy is the current gold standard for diagnosis, it is invasive and fails to distinguish early or borderline rejection from normal functioning, underscoring the need for non-invasive, precise diagnostic methods. Immune repertoire sequencing, which encompasses T-cell receptor (TCR) and B-cell receptor (BCR) sequencing [1], provides in-depth insights into the immune status of transplant recipients. It enables the accurate detection of TCR and BCR clonal expansion, even in the initial stages of rejection [2, 3]. Despite its promise, clinical application has been limited. To address this, we have applied it clinically to assess patients with ambiguous transplant kidney dysfunction diagnoses.

We enrolled 10 recipients experiencing transplant kidney dysfunction with indeterminate diagnoses (ethics approval from Zhongshan Hospital, Fudan University, No. B2022-492). We collected peripheral blood mononuclear cells (PBMCs) for immune repertoire sequencing. Detailed characteristics of the patients are listed in Table 1. Using sequencing results, biopsy outcomes, clinical symptoms, and prognosis, we classified patients into rejection and non-rejection categories with further subdivisions in the rejection group according to rejection type.

**Table 1:** Characteristics of patients

Subject	Gender	Cause of ESRD	Biopsy	Secondary transplantation	Pathological diagnosis	Diagnosis based on immune repertoire sequencing
1	Male	Unknown	Y	Y	ABMR	ABMR
2	Male	Bilateral renal calculi	Y	N	TCMR	ABMR + TCMR
3	Male	Glomerulonephritis	Y	N	TCMR	TCMR
4	Female	IgA nephropathy	N	N	/	B19 + CMV infection
5	Female	Glomerulonephritis	Y	N	TCMR	TCMR
6	Female	Unknown	Y	N	TCMR	ABMR + TCMR
7	Female	Unknown	Y	N	CNI toxicity	CNI toxicity
8	Male	ADPKD	Y	N	CNI toxicity/borderline rejection	TCMR
9	Male	Glomerulonephritis	N	N	/	TCMR
10	Male	Unknown	N	N	/	Infection

**Fig. 1:**
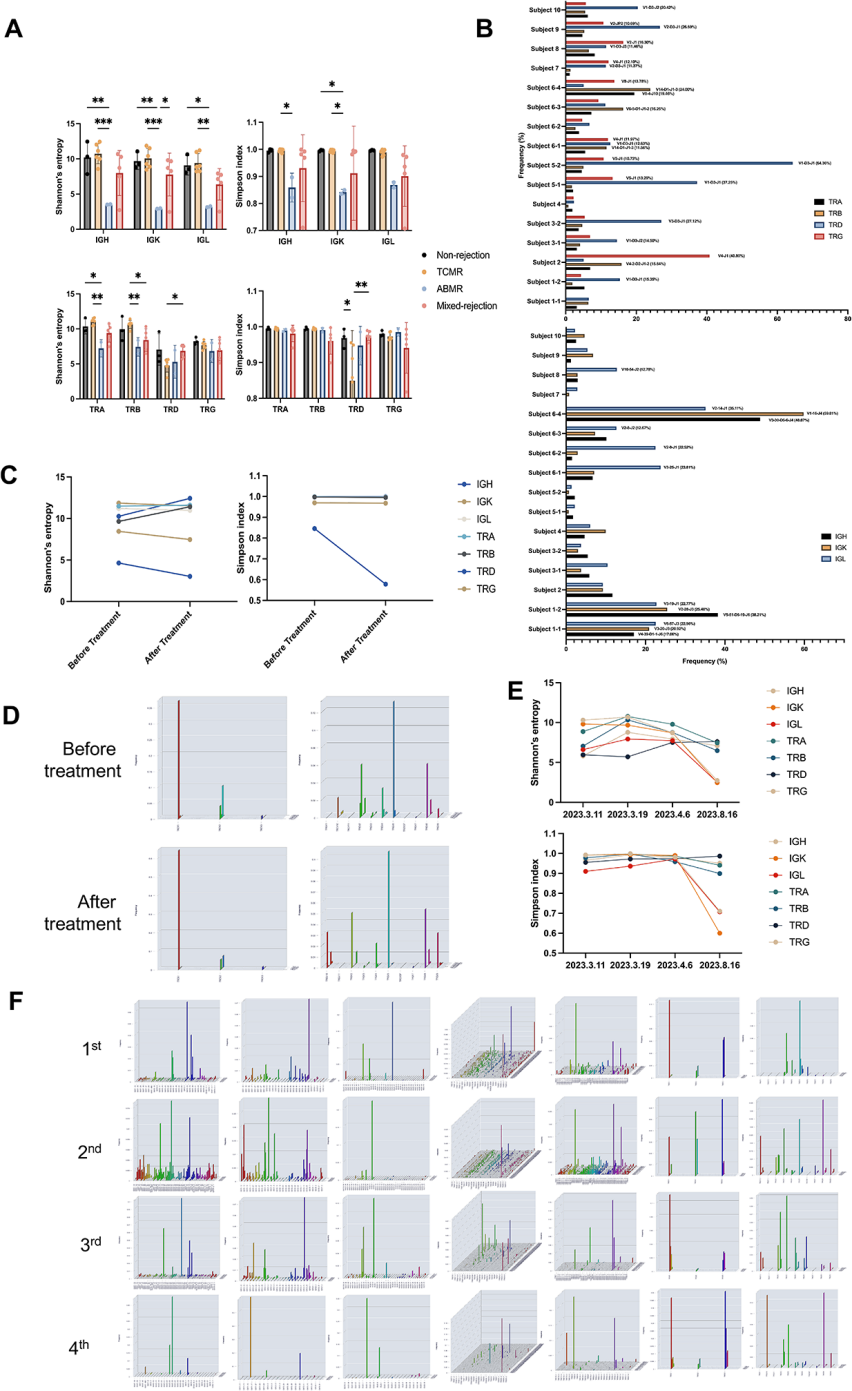
Analysis of the immune repertoire sequencing of transplanted kidneys. (**A**) The clonotype with the highest frequency in each chain of BCR and TCR across all samples from the 10 subjects. (**B**) Comparison of Shannon entropy and Simpson index for each chain of BCR and TCR in the non-rejection group, the TCMR group, the ABMR group, and the mixed rejection group. (**C**) Changes in Shannon entropy and Simpson index for each chain of BCR and TCR in subject 5 before and after treatments. (**D**) The three-dimensional histogram of the expression and changes of clonotypes in the TRD and TRG chains in subject 5 before and after treatment. Among them, the expression of TRD V1-D3-J1 increased from 37.25 to 64.36%. (**E**) Changes in Shannon entropy and Simpson index for each chain of BCR and TCR in subject 6, who suffered from recurrent refractory rejection, across four tests. (**F**) The three-dimensional histogram of the clonal expression and variation of each chain of BCR and TCR in the four detections of subject 6

We assessed the diversity of BCR/TCR in different rejection types using Shannon and Simpson indices. We also tracked major clone fluctuations and highlighted the most highly expressed clones for each chain (Fig. 1A-B). Patients with T cell mediated rejection (TCMR) exhibited an increase in at least one major TCR clone and a decrease in chain diversity. A similar pattern was noted in cases of antibody mediated rejection (ABMR). Patients with mixed rejection displayed abnormal expansion in both TCR and BCR. In contrast, patients without rejection maintained substantial diversity and did not show a significant rise in major clones. We speculate that the increased major clones observed in some non-rejecting patients may be attributable to specific infections that cause kidney dysfunction.

Diagnoses from immune repertoire sequencing largely matched biopsy pathological results, with major clone expansions corresponding to rejection types. Two patients (20%) had different diagnoses via sequencing compared to biopsy. One patient (subject 2), initially diagnosed as TCMR, exhibited significant TRG major clone expansion and was later diagnosed with mixed rejection. Another patient (subject 6), also initially diagnosed as TCMR, demonstrated significant IGL expansion before treatment, indicative of mixed rejection. Following anti-thymocyte globulin (ATG) treatment, the diversity index improved, consistent with clinical improvement. Nonetheless, the persistence of a high-frequency IGL major clone suggested ongoing mixed rejection. A patient (subject 8) with borderline rejection was identified through sequencing, which revealed TRD and TRG major clone expansions, leading to a TCMR diagnosis. ATG treatment yielded positive results. We conducted immune repertoire sequencing on a TCMR patient both before and after the first round of treatment. Post-biopsy TCMR diagnosis led to treatment with basiliximab and methylprednisolone. Pre-treatment sequencing showed significant TRD and TRG major clone expansion with low diversity, confirming a TCMR diagnosis. However, post-treatment sequencing revealed further TRD and TRG major clone expansion, and a decrease in diversity, indicating treatment inefficacy in addressing TCMR (Fig. 1C-D). Consequently, three doses of ATG combined with prednisone were administered, resulting in significant kidney function improvement.

We described a patient with refractory rejection, utilizing immune repertoire sequencing at four time points to track changes in TCR and BCR (Fig. 1E-F). Following the initial rejection, diagnosed as TCMR, serum creatinine levels decreased after ATG and steroid pulse therapy but subsequently rose rapidly. The immune repertoire indicated a high abundance of the IGL, hinting at an early potential for ABMR. Subsequent treatments with plasma exchange, IVIG, and rituximab led to improved TCR diversity. However, a severe pulmonary infection precipitated a second rejection episode, characterized by diminished TCR and BCR diversity, indicative of mixed rejection. Upon recovery, immunosuppression was adjusted to CsA + MMF + Prednisone. A subsequent increase in serum creatinine levels necessitated IVIG administration and sequencing, which identified elevated BCR and TCR clones, signaling mixed rejection. Treatment with methylprednisolone resulted in reduced creatinine levels, culminating in a favorable prognosis.

From a clinical standpoint, we delved into the potential of immune repertoire sequencing as a precise diagnostic tool for transplant kidney rejection. Our research uncovered that by tracking shifts in immune repertoire diversity and the frequency of TCR/BCR clonotypes, we can identify the onset and type of rejection, including borderline changes. Furthermore, its sensitivity to immune repertoire fluctuations enables us to monitor treatment effectiveness, offering a clear and insightful view of the patient’s condition post-treatment. It also offers distinct benefits in diagnosing refractory rejection. Additionally, our study revealed changes in the immune repertoire of patients with viral infections or calcineurin inhibitors toxicity. These changes are marked by a relative uptick in certain TCR [4] or BCR clonotype frequencies, while the overall diversity remains mostly stable. Nonetheless, definitive patterns in these instances necessitate further data for conclusive analysis.
